# Responding to Healthcare Disparities and Challenges With Access to Care During COVID-19

**DOI:** 10.1089/heq.2020.29000.rtl

**Published:** 2020-04-14

**Authors:** Ana Núñez, Maria Madison, Renata Schiavo, Ronit Elk, Holly G. Prigerson

**Affiliations:** ^1^ College of Medicine, Drexel University, Philadelphia, Pennsylvania, USA.; ^2^ Heller School for Social Policy and Management, Brandeis University, Waltham, Massachusetts, USA.; ^3^ Mailman School of Public Health, Department of Sociomedical Sciences, Columbia University, New York, New York, USA.; ^4^ Founder and Board President, Health Equity Initiative, New York, New York, USA.; ^5^ Department of Medicine, Division of Geriatrics, Gerontology and Palliative Care; University of Alabama at Birmingham, Birmingham, Alabama, USA.; ^6^ Center of the Southeast Institute for Innovation in Palliative Care, University of Alabama at Birmingham, Birmingham, Alabama, USA.; ^7^ Cornell Center for Research on End-of-Life Care; Weill Cornell Medicine, New York, New York, USA.; ^8^ Sociology in Medicine, Weill Cornell Medicine, New York, New York, USA.

Viruses know no boundaries, but the impact of pandemics highlight faulty health systems and at-risk individuals. The novel coronavirus of 2019–2020 pandemic has hit certain groups of people within the United States more severely than others. Those living in underserved areas, often with financial hardship, and black and brown individuals, are more prone to experience sickness and death from the virus. This roundtable discussion brings together several experts from various fields related to health equity to address these disparities and recommend actions needed to attain equity.

***DR. ANA NÚÑEZ:* I recognize that identifying one top ranking priority is a challenge, as there are many. That said, what is your top-ranking priority during this current COVID19 pandemic? What item most needs to be realized or addressed?**

**DR. RONIT ELK:** My number one priority is how culture influences and fundamentally shapes how people make meaning out of illness, suffering, dying, and death are, and how culture strongly influences people's response to getting a diagnosis to an illness, as well as treatment preferences.

And the problem is that we have a lack of appreciation in the U.S. for the cultural differences that may and in fact do compromise care for seriously ill minority–I call them patients, but people. And that is my number one priority.

**DR. MARIA MADISON:** Thank you so much, Dr. Elk, for your comments, because my number one priority is fairness. When I think of fairness, I am thinking of investing in equitable access to prevention, mitigation, and treatment for COVID. That includes looking out for the most vulnerable populations and their needs for basic things, including clean water. When we say prevention is easy, that we should wash our hands for 20 seconds, it is easy to forget that a large proportion of the population in the U.S., if not the world, does not have access to clean water, or the Internet access to learn about best practices or threats to our food supply, but even more importantly, in order to assure that we try to protect our frontline workers, personal protective equipment (PPE) supply chain, investing in vaccines, or even investing in evidence-based treatments. So for me, the number one answer has to focus on promoting equity for all the reasons Dr. Elk just described. Right now, we are seeing that a disproportionate number of people who are suffering and dying from COVID-19 are black and brown folks. We can dig deeper into why that is when we get to the other questions, but it really draws attention to the inequity in our society that allows us to continue to oppress populations that are already at risk for all aspects of inequity.

**DR. RENATA SCHIAVO:** My number one priority is to protect marginalized and vulnerable populations from this pandemic and beyond. I feel pandemics have this very strong way of showing us how widespread social and health inequalities are, and how in the day-to-day they prevent people not only from protecting themselves, but also from leading healthy and productive lives. Within the realm of vulnerable and marginalized populations, there are three main groups that come to mind as examples. First, there are children who live in poverty who may not be directly affected by the serious health consequences of COVID-19, but in many cases depend on being in school for their only meals. The pandemic has shown that in addition to school-based nutrition, we need to think of additional systems to deliver child nutrition to the 20+ million children who live in poverty in the United States, especially in communities of color that have been marginalized and discriminated against for centuries, and experience high rates of poverty. The Family First Coronavirus Act has tried to address these issues, but it is just a drop in the bucket compared to the needs we are facing.^1^

The second group is the homeless population. We talk about social distancing and washing hands. These are very difficult behaviors for people who live in shelters or in the street. We have seen communities coming together and trying to install temporary sinks. But again, we need more comprehensive interventions.

And third, of course, the communities of color who are more significantly impacted. We are already seeing that the highest mortality is within black and brown communities. This has to do with a history of lack of investment in communities of color, poverty, racism–please let's use that word, and a variety of other issues that have determined a higher burden of health and social inequities. I will stop it here, but again, it was very difficult to talk about one priority, so I decided to make an example of populations within the ones that we need to protect.

***DR. NÚÑEZ:* Thank you.**

**DR. HOLLY PRIGERSON:** I direct the Center for Research on End-of-Life Care at Weill Cornell Medicine. Although my focus is on disparities, I have concentrated my research mostly on psychosocial influences on the poor or inadequate care at the end of life. My focus has been on mental health issues and how patients and families might feel abandoned, as well as how poor communication and lack of resources to attend to psychosocial needs are often overlooked in life-threatening illness, especially for marginalized patient populations and families. I look more at the intersection of how psychosocial influences might be affected and impact access to care, receipt of care, feelings of injustice that some people might have gotten a ventilator whereas other people may have been denied a ventilator. We are trying to leverage the available resources that we know exist to try to remove barriers to better care for everyone, but by targeting a lot of the psychosocial issues that we think really account for who gets what.

***DR. NÚÑEZ:* Augmenting much of what you all said, the area that I see and feel that is the priority from my perspective is something that sounds sort of simple: process. There is not a coordinated, integrated, thoughtful process right now. Instead, we have this patchwork of competition where every institution and individual has to figure it out independently.**

**The fact that there is not a coordinated and integrated approach that oversees this pandemic speaks to the fact that we only have a public health finance structure. We do not actually have a functional, robust public health infrastructure. This void increases adverse health risk for vulnerable, marginalized populations and stresses these populations and the system. It's very difficult to ignore this impact now because we are seeing the exposure of vulnerability–risking mortality rates is just one marker–which really speaks to the pressing issues of need for integration and coordination of public health and prevention. Ultimately, it will affect everybody.**

**The question is, is this our opportunity to take this challenge as an opportunity to do things differently, or do we go from here and not make the needed changes that can put us all in a better position?**

**DR. PRIGERSON:** By saying “process,” that sounds more macro, conceptual, zoomed-out kind of approach. And I think related to zooming out, I would say communication is important as well. By “communication,” I am referring to barriers to effective communication to decision-making, to getting care, to receiving care, to working with families at home to connecting families with work and employment. It is communications about medical decisions but also more mundane responsibilities such as going to shop for food and pick up cleaning supplies and all the sort of ways in which this COVID-19 has affected our lives.

Communication, and in particular, telecommunication appears to have become a normal part in the way of life for many of us. Right now, this is how we are all communicating with each other, but it is important to bear in mind that access to telecommunications for certain communities might not be available. Maybe health literacy might affect communication and understanding of medical choices before making decisions. I think communication is a key aspect in how this pandemic has adversely affected different communities.

**DR. MADISON:** Yes to “process” but it must be fair process. As of the time we are having this discussion, we are seeing states like Alabama and Washington creating triages that some are calling ruthless utilitarianism, because they are singlehandedly creating hospital practices with a process that may be likely to inhibit treatment and care for certain groups, again, including our most vulnerable populations. That is why we are seeing high rates of morbidity and mortality for black and brown folks.

**DR. ELK:** So most of us have defined the problem in terms of what is happening. I would like to discuss, when we get best practices, what happens in the hospital, because this is where the physicians and nurses and ethicists and so on, have to do. I will talk about how to make advance care planning decisions and how to incorporate leaders. My expertise is with African American communities. You must incorporate pastors in your ethics committees. If you do not do that, you could be doing something morally wrong.

In the United States, they do not give sufficient respect to the pastors, who are not only leaders in terms of faith and spirituality, but in terms of everything else.

**DR. SCHIAVO:** I want to add something to what Dr. Prigerson was saying about the importance of communication in this moment. Some of the main principles of communication, and especially risk communication, are trust, transparency, and community engagement. A lot of communication has not been meeting these principles because it has been conflicting. It has not included community leaders who actually have the real understanding of the communities for which this communication is intended, who are trusted sources. Often these are the individuals who really know best about the needs, preferences, and priorities of specific communities, such as communities of color, children, and other populations. We need really to think of risk communication not as the remedy when things go wrong, but something we should be prepared for in advance, during inter-pandemic times.

***DR. NÚÑEZ:* Currently we are overwhelmed by news and social media activity. An important question for me is, how do vulnerable populations identify trusted sources of communication?**

**DR. PRIGERSON:** We are actually trying to develop what we call a “divine intervention” that capitalizes on the trust that we have found that healthcare chaplains and hospital chaplains have, particularly among our black patients with advanced-stage cancer. In our studies, what we have found is that for whatever reason – we do not know the mechanisms – but when very sick, dying patients seek a hospital chaplain, they are more likely to sign a do not resuscitate order. They are less likely to die in an ICU. They are more likely to enroll earlier in hospice. We have been trying to understand this, but we suspect it has to do with feelings of trust that may lead to an enhanced ability to communicate. Leveraging the power and influence that healthcare chaplains have in this crisis might be effective. We think this may be a missed opportunity, because healthcare chaplains have the ability to understand where people in their communities are coming from, as well as talk the talk to physicians.

**DR. ELK:** There are two things I think are key and that may be promising aspects. One is in terms of transparency and reaching out to the community. At the University of Alabama, Birmingham, I have been involved with the School of Public Health, where we have reached out to the black pastors, housing authority and the dean of the medical school, who himself is black, and have set up a series of webinars.

The first webinar was on Saturday, April 6, 2020.^2^ There were 1,500 people on the webinar. Many of the people were from the housing authority, just regular people. Many others were black people from the community. The whole point was to discuss all these issues.

There were specific goals with this webinar: one was to help people in the community understand how this illness progresses and so on, and our dean did speak about that. A little bit too much jargon, but okay. And then, the other goal of the webinar was to educate participants on how to protect themselves.

I am hoping that I can partner with the housing authorities and we can create culture-based messaging, because creating messaging that the white man and the white middle-class has developed for the large audience does not work. We know that. So I wish we would stop doing that. And so we did create culture-based messaging, and we are going to continue to do that.

What I am begging hospitals to do is to include leaders who are black, alongside other ethnic groups, on ethics board before making decisions on protocol for care allocation. Failing to do that is failing to care about the black population and can lead to lawsuits, which is discussed heavily if bottom dollar is what the institution cares about. I do not care if you have already got a policy. Change it. Revise it.

The other thing is, there is a lot of pressure in the United States for having a written advance care directive. That is not going to work in the black community, particularly in the South. If I sign something saying, “Do not resuscitate my mother, you will do nothing. As it is, you do nothing for our people. Why should I sign a piece of paper?” So do not pressure people.

And if I say that culture influences how you make decisions, in the black community in the South, it has been shown that millions of people have held belief in the concept of, “There could be a miracle. God can make a miracle happen.” I do not know why people have such difficulty with this, since so many people read the Old Testament, where the word “miracle” is everywhere. But it is very, very difficult for physicians to understand this concept. If a miracle can happen, then you have to do everything that you can for the patient until they decide.

Another thing is that nobody dies alone in the black community. There will always be a pastor or somebody who will sit with them. In this time of COVID-19, we have people sitting alone. There are a couple of practices that I have seen that could be used. One thing that can be done is to have someone sit with the patient, remembering that this is somebody's loved one. Yes, it may take hours, and yes, there are other patients to care for. But there is always someone who can sit there and be with the person and pray with them and maybe sing a church song with them. That act can be tremendously meaningful.

The other thing is funerals. We know what happens at funerals. It is not only blacks. In Israel, they have had the ultra-Orthodox, who went to funerals where there were thousands of people. The percentage of the virus in that community is very, very high.

What can we do? One way is to have the funeral through Facebook, where singing and praying can be facilitated through a virtual platform.

I also heard from a pastor who was talking about his grandfather who passed away from COVID-19. His grandfather was a very, very senior, very respected black pastor. Thousands would have come to his funeral, but could not because of the virus. Instead, they had five people go into the church and record music. Five people somewhere else recorded the sermons. Then they put it all together and people from the church drove up and were able to see the whole video they created this way.^3^

**DR. MADISON:** I want to prevent the deaths. I want us to think about why 70% of COVID-19 deaths in places like Chicago are black folks. I want the country to take a public health approach, which starts with prevention.

In terms of prevention, the reason why we are seeing these rates of morbidity and mortality is because of rationing. The reason we are seeing rationing is because of the lack of planning on the national level, the lack of taking lessons not just previous pandemics and plagues, but also from other countries that have already determined and discovered. For example, cordon sanitaire does not work, so we need to do physical distancing. We need to promote social connections so we can promote sociobehavioral health and well-being.

When we do not put ourselves into a situation where we have to ration, we should see fewer deaths, and we should see much less suffering, particularly in vulnerable populations. I think the fundamental problem is that we have gotten into a situation where we have to ration.

So why are black and brown folks dying more? Is it because of rationing of ventilators? Is it because of rationing and not providing an environment where there is equitable access to treatment, equitable access to screening? We have to look at the entire chain of events that happens. It begins with prevention, and making sure we do not have to get into a situation of rationing. Rationing is one of the root causes of why we are seeing this demographic differential in morbidity and mortality. When it comes to states determining who is the most worthy, who should be the first in line to have access to screening, treatment, ventilators, it is not our black and brown folks who are already immunocompromised, who are already in high-risk groups from obesity, diabetes, and hypertension. We need to do whatever we can to reduce this pandemic of rationing. We need to promote case finding in all populations. We need to promote contact tracing. We need to promote screening. We need to learn the lessons, not using cordon sanitaire. It reduces trust. It does not promote communication. We need to increase fact-checking and transparency in reporting on who is most infected versus affected.

There is so much we could go into related to the CARES Act and joblessness and the relationship between joblessness and comorbidities, for example.^4^ But I saw this one data point that described how for every 1% increase in unemployment, it leads to a 3.5% increase in opioid addiction.^5^ The pandemic's economic effects alone will exacerbate our drug and mental health problems down the road.

**DR. PRIGERSON:** We are developing a lot of psychosocial interventions that deal with the opioid epidemic and how people are responding to this pandemic psychologically, especially when they are unemployed and at home and life looks hopeless. Alcohol sales have skyrocketed.^6^ People are going to self-soothe, and that is going to cascade to a whole bunch of problems down the road.

But that is not what I wanted to react to. What I was wondering, in terms of process and in terms of equity and thinking down the road, is what happens when people feel that there was an unfair distribution of who got saved, who got the ventilator? What could be done now to have more transparency in the ICU to support decision-making?

There was recently an editorial by Daniella Lamas in the *New York Times* about the decisions and the criteria for deciding which patients would benefit from getting a ventilator and which would not.^7^ Ironically, or paradoxically, in end-of-life care, there are always recommendations like, “Do not put an advanced cancer patient on a ventilator anyway. It is futile. You are wasting valuable resources, and it is burdensome, and they are not going to survive.” So that is not a good use of a very scarce resource.

How can those rules about who should live and who is going to be put on a ventilator be made more equitable so to address the concern down the road, so there are not lawsuits saying, “You discriminated against equally needy patients who would benefit from scarce resources but denied care for other reasons.”

**DR. MADISON:** A real quick answer goes back to what Dr. Elk had said before. Your triage committees need guidance documents, and those should be written by ethics committees that are representative of the community. So you should have black ministers involved. You should have every demographic and profession included in your ethics committees to collaborate in creating these guideposts so that it is not left up to an individual, implicitly biased practitioner.

**DR. ELK:** And having just one black person is not enough. That is insufficient.

***DR. NÚÑEZ:* At least from what I am hearing from other physicians, they are desperate for these equitable protocols. In the absence of protocols, clinicians are having to make the decision in the moment with so many things happening in terms of the trauma, lack of PPE, and so on and so forth. Healthcare providers are desperate for these equitable protocols.**

**And I will just remind you that we know data that say, if you have a committee, and 30% of that committee is not representative of the population, you do not have voice. So it is important when we are looking at these committees that it really is that percentage of the committee to bring that voice into the equation. But I will tell you that equitable protocols are desperately sought by healthcare providers who are in the trenches having to make these decisions, and with no time, on top of being under-resourced and potentially unsafe.**

**DR. ELK:** There is a hashtag on Twitter, #pallicovid, used by the palliative care community, linking to all kinds of resources that can help. The reality is, probably only the palliative care physicians or clinicians are looking at it, but others need to as well, because this is the group that has the expertise.

Now, unfortunately, even though palliative care is the group that has this expertise, they do not and have not been trained in determining cultural aspects of care. That is why a paper on cultural aspects of care, especially at end of life, is so, so key.

*Health Equity* just published my article and in it we include a table with information about the differences in approach for how to talk to southern black and white patients.^8^ And who determined that? The community members, both black and white. Everything that is in there is a cultural guide for clinicians. Now, this was done in the rural South. I have no idea if it works up North. Dr. Prigerson and I are going to be collaborating on another study to see to what extent that works up north. But at least, if you respect what the community has asked, then you will go a very long way in showing respect, which, in turn enhances trust. And those additional suggestions of having somebody there with a black person, when they are ill, using FaceTime so the family can sing and pray with the patient as they are dying. How difficult is that? It is not difficult. It can easily be done. You want to build trust? Do that.

And by the way, I do not believe that it is a waste of resources, particularly for people whose culture believes God can create a miracle, and if that is what the family wants and believes, then we should respect the patient and/or family's values and act according to their established goals of care. It does not matter if medically it seems like, “Oh, this person will live and this person will die.” You have to be equitable, as Dr. Nunez says.

**DR. SCHIAVO:** The issue of preparedness is of the utmost importance. We definitely need more preparedness for a variety of different issues. For example, I published with co-authors a systematic review, which perhaps is still the only review on communicating risk in epidemics in low- and middle-income countries, and also includes eligible studies on marginalized and at-risk populations here in the United States.^9^ Some of the things we have been discussing resonate with the findings of the review, which point to the importance of communities and community engagement. As supported by several studies included in the review, when community members and families were involved, communities or patients were also more likely to adopt and embrace mitigation measures.

Another lesson that we learned from Ebola: we cannot go into communities and tell them to suspend traditions for burials and funerals during a time of crisis. We need to think about culturally sensitive rituals to substitute for existing traditions during the preparedness phase. So again, the preparedness process is really key.

I'd like to change the topic. In addition to engaging community leaders in finding solutions for issues related to the rationing of scarce resources (for example, the use of ventilators or protective equipment) so that we prioritize vulnerable and underserved populations, especially communities of color, professional associations representing the black and Latino communities should also become involved. These associations should consider issuing guidelines that physicians and nurses desperately need to treat and prioritize patients who most need these resources, especially in disparity settings.

Finally, I had prepared something on paid leave, because among the most promising changes that I see happening–that, again, is not sufficient to meet the actual need–is the Family First Coronavirus Response Act, as related to the provision of paid leave for at least some of the workers.^10^

I am not an economist, but unfortunately, this provision is really a drop in the sea, because we know that up to 19 million people will be excluded from this provision.^10^ On the other hand, people need to stay home as a way of protecting themselves. But we know that a lot of people are excluded from the paid leave provision, and would need to choose between protecting themselves or paying rent and putting food on the table. And this happens primarily within the food service industry and other industries where the people are really on the front line of the epidemic, and/or where workers are from communities of color, or women, or from other vulnerable populations.

Although the Family First Coronavirus Response Act is a step forward, we need to engage communities to make it more of a reality for all Americans, because paid sick leave is something that is important not only during this pandemic, but it actually is a human right to be able to take care of one's health and the health of others during times of crisis and beyond. So, it is a step forward, but it is a fraction of what we need.

***DR. NÚÑEZ:* We talked about isolation, opioid use, and issues related to mental health. I think it also bears mentioning that issues of intimate partner violence go hand in hand with alcohol use and gun sales. It is also worth recognizing that both morbidity and mortality, for women, is also likely to explode during this pandemic. Many of the places that are being serviced that support victims of intimate partner violence are not-for-profits that receive federal funding, and these are going to be places with incredible need, especially in a time where there is isolation, alcohol, unemployment, et cetera. It bears mention.**

**The most uncertainty in terms of the future of preserved food supply, ways of moving forward to continue living, are all on the backs of populations that are predominantly the ones that have been most discriminated against, or who suffer and bear the burdens in terms of inequities. These are not necessarily the ones who have free access to be able to get on the Internet to find information. And even if they do, to other people's points, it is in such a high level of jargon, or written in English language when the individual maybe doesn't speak English. We need much more profound translations of that content to support health literacy as a way to get messages out for everybody, from pastors to communities, because one of the challenges that I see as a physician is that suddenly everybody is interested in science.**

**Science is not perceived as being irrelevant anymore. Science is not a ridiculous thing. Science is not something that people do not need to know about, and suddenly people are wondering, “What do you mean by immunity?” I think this is fabulous, but I am not sure that those messages are effectively reaching the communities of need. We need to be able to make those connections and parlay into building trust. Currently, there are too many mixed messages.**

**DR. ELK:** One of the things that you said about community is key. One of the research methods that is very, very appropriate, however extremely difficult to do in a very tight timeline, is community-based participatory research, where you could partner with the community. To reach the communities, we have to work in partnership with them. We can do a community-based research project where we can develop prevention guidelines in words the community will understand and according to the community's values. Even if we can only determine feasibility, the goal will be to help save some lives. But if we can determine that this is a method that we can do when the next crisis comes, we will be ready to have such studies.

And to add to what Dr. Schiavo said, I would like to stress, we have to learn from what happened in the Ebola epidemic, when whites were attempting to provide aid, they did so without paying respect to the culture. They did not listen to the people experiencing the health crisis. They did not incorporate the culture of the people into it. If you fail to incorporate the culture of the local people, you are doomed to failure, and more people will die.

It is a matter of cultural humility that people have to learn, and especially physicians, who unfortunately do not effectively receive this as part of their training. It is part of the nurses' training, but it is not part of the physician's training.

***DR.NÚÑEZ:* I agree with you. I think that with the “do to” rather than the “do with,” mentality, especially with the scarcity of key equipment right now, there is a propensity to say “Let us do something.” This results in, “Here is the shortcut that lets us something.” And I think that the best thing to do, even though it may take a bit more time, is actually reaching out and including community networks to create a better outcome in the end if there is a matter of trust.**

**DR. SCHIAVO:** Actually there is evidence also from the Ebola crisis such as for example some interesting case studies on Sierra Leone and Liberia from UNICEF showcasing that when communities finally got involved, not only in research and intervention design, but also in the implementation and the evaluation of solutions, and in building trust in the community about the recommendations for protection, finally, the Ebola epidemic subsided.^11,12^

And I think this is a very important lesson, especially because in the United States, I feel we do not integrate enough community engagement in intervention design, implementation and evaluation. We have imported to a certain extent the community health worker model, but for the most part this model is being implemented in a very limited way because we primarily train people to disseminate information that experts designed. It is not really the same as the kind of community consultative process we need, especially in moments of crisis when we really need to empower communities, giving them ownership of solutions.

**DR. MADISON:** There is a long list of steps that countries such as Taiwan, Singapore, and others took that both flattened the curve faster, and reduced the prevalence of disease and mortality.^13^ In that long, wonderful list is the item that they addressed the issue of disease stigma and compassion. I do not know where that exists in our state-by-state plans. We do not really have a national plan.

But that was listed as a policy in Taiwan and it made considerations for those affected by providing food and frequent health checks. It also included encouragement for those under quarantine. And the rapid response included hundreds of action items in their supplement. But just imagine including disease stigma and compassion as a part of the process.

**DR. ELK:** Andy Slavitt is one person who has shown unbelievable compassion and action. He was the head of President Obama's Medicare and Medicaid CMS, and helped develop the Affordable Care Act. He is very knowledgeable and very connected. He has taken it upon himself to develop an organization called the United States of Care.^14^

Andy Slavitt has put together so many initiatives. For example, he set up a site where ventilators can be shipped from one place to another, even before there were other initiatives. His group also set up a step-by-step guide for bringing resources to underserved communities.^15^ (See Appendix S1; © United States of Care Campaign and reprinted with permission.)

So you can take it into your state, and all you need is the governor of that state, for example, to follow this step-by-step guide. Now, let us see how many states use this incredible model.

**DR. PRIGERSON:** In terms of resources, I do not want to plug too much of what we are doing, but we are developing online resources to aid communication between families, between families and medical professionals, particularly in the life-threatening ICU situation. But also we are developing tools to prevent people from dying alone and funerals not being able to happen in accordance with and culturally specific cremation and burial practices.

We are developing an app that actually is a virtual memorial. We have developed something called the Living Memory Home to help families in that time–it is not going to be a substitute for actually convening and having face-to-face ceremonies at some point, but the idea is, I think people are really struggling with this forced separation and lack of communication as everyone–everyone is essentially a shut-in right now. We are all shut-ins.

And when your loved one is dying in the hospital, and you are shut in, regardless of your race or ethnicity, you are upset, you are frustrated, and you need tools to help you communicate better and more effectively.

The other point I wanted to make in listening to everyone's' great suggestions, is that all our suggestions are essentially top down. Community-based participatory research is great in that a lot of voices are heard. What should recommendations to actual families do? What should be some of the simple patient prompts or family caregivers' checklists for things that they should do to help them protect themselves and ensure their interests? We are always thinking about how we can help other people through being very instrumental and telling them what to do or treating people differently. What can they do themselves to have their rights and interests and values respected?

***DR. NÚÑEZ:* I certainly read that in some places patients actually got an iPad that is covered in plastic so that they are not alone, they can connect with somebody and so on and so forth. And if we are talking about best practices, whether it is a phone or any other kind of device, to support that connection when someone is critically ill or at the end of life, that is as instrumental as having an IV.**

**Now, granted, I would submit to you that in terms of dying in a hospital, dying alone is a very frequent thing and very culturally devoid thing outside of that hospital. And so perhaps this is a practice we need to bring in that, just like the IV, there is this digital access to music, to a spiritual advisor, to family, to singing, whatever that is, that as a person surrounded by all the illness in a hospital, they do not necessarily feel alone.**

**As long as you attach it to something, it is not like you are mandating that another person necessarily be there, but if an IV is essential, then perhaps we are saying this as well, because we need to pay attention to the humanity of individuals as they go through this struggle.**

**DR. MADISON:** I think that what communities also need to do, particularly my community and black communities and international communities, is to destigmatize accessing mental health services. By destigmatizing access to mental health services, society also has to provide free services, right? So let's promote access to free telehealth, promote access to paid sick time if you are fortunate to have a job, and promote access to free testing and treatment. We should promote destigmatizing access, whether it is for behavioral health or clinical health care. But right now, some of the barriers to access are both inside and outside of the community, so we should somehow support bridging that.

In Massachusetts, the Department of Public Health, through the Massachusetts Public Health Association, highlighted four action items. And I believe that the fourth one is the one we have not mentioned yet, is so important because it adds to an increase in prevalence and incidence of this pandemic, of COVID-19 pandemic, and it is to enact a moratorium on evictions, foreclosures and termination of public benefits.

What can the individual do about that? Not much. If you lost your job and you call up unemployment assistance, you are not even able to get off the waitlist on that phone. People are waiting two, three weeks to get a response in order to get unemployment insurance. I want to also stress that people finding themselves unable to get through to a representative at the unemployment office should document their every try.

And so in the process, you are getting someone telling you have to be evicted from your housing, and foreclosures, and losing your public benefits. So it is ecosocial theory. It is all around the lifespan of what is happening to our most vulnerable populations. Some of what we can do within our group, within our community is to destigmatize access and promote lobbying and advocacy. But it really is oppression working through ideological, institutional, interpersonal, and internalized mechanisms.

**DR. ELK:** One of the things that Dr. Prigerson had raised was, what about doing something for the patient? There is a tool that was developed at UAB in which the palliative care doctors said, “We will sit with your patient.^15^ Tell us…”–they have developed a little questionnaire. The patient's loved ones fill in the answers. “What does he love talking about? What is important to him? What is the name of…?” This is meant to help the practitioners get to know their loved one, the patient, intimately, and can help to represent the loved one in a very unique, individual way.

There is also a tool for providers on how to communicate at this time, developed specifically for COVID. All of that is both on Twitter under #pallicovid, and also on Facebook, which is much easier, and is COVID-19 Palliative Care Providers. It is open for anybody. I saw that 3,000 people are already on the Facebook group as of the time of this discussion.

It has all these tools. If the physicians and others are looking for tools, the palliative care people have the perfect tools. One of them deals with how to communicate and what to say. They use a lot of acronyms to help clinicians remember them.

**DR. SCHIAVO:** I want to highlight something that has not yet emerged from the last discussion, which is the digital divide. We all talk a lot about digital health, we talk about those apps. But these media approaches are not necessarily going to reach the vulnerable populations we need to protect, where word of mouth, community gatherings, churches, and similar channels and venues are still the preferred ways of communicating. We are already seeing that when school went online, some schools in disadvantaged neighborhoods were left scrambling to figure out how to provide online instruction.

In addition to this, we are in the middle of an infodemic, and there is a lot of information and misinformation out there. Easy and widespread access to social media, which we did not need to care about during H1N1, really have a prominent role in disseminating this misinformation. And although some of the vulnerable populations may not use social media as their preferred media of choice, they hear from other people who have read things on social media.

So we need to be aware of these challenges and prepared to equip the public health infrastructure to react to hoaxes and misinformation. I was reading the other day that there were some hoaxes in Africa saying that blacks were not susceptible to COVID-19.^16^ We need to be prepared to counteract misinformation, and the only way to do this is having, again, governments and public health agencies to work with community leaders, so that those leaders become our rock stars on social media and within other information settings.

Let's give them social media accounts. Train them to use social media. Let's do something that actually brings their trusted voices to the communities they reach, because whether these communities are on social media or not, they hear from others who are on social media. This is also another important aspect that may have an impact on training of the public health workforce and on the overall infrastructure.

***DR. NÚÑEZ:* I want to agree with you. I mean, I will share with you that in terms of our community participatory research project, Philadelphia Ujima, we brought in the radio celebrities, because the radio celebrities are important from a cultural perspective with lower-income residents in the city. Some of these radio celebrities had profound credibility, and whatever they said was viewed as true. Unfortunately it is the case that right now there is no way to certify what is actually credible information.**

**Dr. Prigerson raised the point that we have talked about a top-down approach, and Dr. Madison eloquently talked about how there is so much in terms of the infrastructure that does not exist, and top-down is important. But I think that some of the bottom-up is, how do we attend to the legitimate disenfranchisement of our at-risk populations, many of whom are saying, “You do not really care about me. I am expendable. I can clean. I can pick in the fields for your food, but I am not going to have time off, and if I am a casualty of this pandemic, well, then, you do not really care. Somehow I am supposed to continue to be engaged, maybe vote, and to be part of this process. How does it make sense when it seems that you all do not really care about me?”**

**And so I think that there is a component that we have to reach to address that legitimate disenfranchisement as well as figure out through culturally competent sort of communication about how can they have some agency in this, how do they recruit help for when their loved one is sick, identify who were all the individuals that need to be in the loop on that conversation? Negotiating the health access process is difficult for most of us, even in better times. How can we streamline the process, provide navigation help during this global pandemic?**

**I think that disenfranchisement linked with health, health literacy, and misinformation, or “the infodemic,” is an important part of the storm, and if we do not address that, no matter what happens top down, the disenfranchisement may very well explode.**

**It is important to mention that disenfranchisement is a useful way to control the populace, because if everybody is looking at everybody else, the problem is always going to be that other person. It is us and them. The community affords strength, innovation, and cohesiveness in coming together to find and promote solutions.**

**That being said, we are hearing in the media lots of amazing examples where people are coming together to form community. This is too often drowned out by the sensational stories of hoarding and price gouging. We do not hear the common acts of checking on the elderly neighbor and sort of going grocery shopping.**

**We do not hear about that, because, again, that does not sell eyeball time for the evening news. It is important that we think about how to best use community-focused, inclusion-promoting messages as one of the antidotes to the infodemic.**

**DR. PRIGERSON:** In response to all this, and the infodemic, we are developing some tools. We call them GIST, “Giving Information Simply and Transparently,” so that when oncologists talk with advanced-stage cancer patients, you are disenfranchising patients if you talk about millimeters of tumor growth, or you talk about drugs for which they do not understand the mechanisms of action.

We are developing this intervention to both address the infodemic, to simplify and clarify main talking points, and insist that physicians have patients leave a clinic visit doing what in psychology they call cognitive interviewing, ensuring that patients have enough information to make an informed choice–they do not need to know every single fact, but maybe the physicians or the medical community needs to decide what are the main kernels of medical information that, without which, anyone, regardless of race or ethnicity or language or education level, needs to know to make a choice that will resonate with them, that will be consistent with their values, and consistent with informed values.

So we are trying to reduce disparities, but through education and information, both on the parts of having physicians communicate to empower patients to have the information they need, to insist on the care that might be consistent with what they would want.

It is always awkward to say what patients would want. My husband and I always argue about goals of care. No one's goal of care is to die well. You know? No one wants any of this. But as researchers, we cannot fix every problem, so what we are trying to do is at least level the playing field in terms of equity and addressing the infodemic. There are too many moving parts and complications to really have a grasp of what you need to know to get care that you deserve.

***DR. NÚÑEZ:* In summing up our conversation, what are the best words of wisdom you would put out there?**

**DR. MADISON:** We must take a holistic approach, top down, bottom up, including all voices, and promoting agency to address particularly the most vulnerable. If we can address issues within the most vulnerable populations, we address the entire population.

**DR. SCHIAVO:** I would like to say that pandemics have a way of showing us how much we are interconnected. Taking care of everyone in our communities and being our brothers' and sisters' keeper is not only an important human rights issue but also benefits the health of everyone. I hope that this lesson is not going to be forgotten too quickly, as we have seen so many lessons be forgotten in the past. As Dr. Madison said, we really need to address all the social and political determinants of health so that we can advance health equity and racial equity in the years to come and protect people during this pandemic. In the meantime, I would like to ask for everyone to take the time to thank the people who are on the frontline. Yes, the healthcare workers, but also the food and pharmacy cashiers, the sanitation workers, the hospital housekeepers and cleaners, and everyone who puts their life at stake every day, so that so many of us can stay safe. I think it is important to say thank you, because a lot of them are making huge sacrifices for the common good, and we need to do our share and at the minimum to thank them.

**DR. ELK:** In terms of prevention, please, work on partnering with communities to develop prevention messaging that is not as complicated as what's on the CDC or other health care sites, but is instead very simple, and not only that, takes cultural differences into consideration. Just a photograph or a picture of a Native American person or somebody in some tribal dress is absolutely insufficient.

A lot of nurses already have training in cultural competency, but sometimes physicians don't have quite as much competency in this area. It is important for all practitioners to show cultural humility and to not talk down to patients, but to ask what their cultural values and preferences are. And then once you know them, respect them. And if you do not know, there are tools and guidelines to improve cultural competency. Look at the article that we have just published in *Health Equity*.^8^

**DR. PRIGERSON:** One thing that struck me as a way to synthesize what we have been talking about is the huge importance of communication in making sure that families are connected, that health professionals are connected, that people get adequate information, that people's preferences and needs are heard. It all depends on facilitating and improving communication between patients, families, communities, and the medical team. There is hope, but we also know that a lot of work needs to be done to improve not just access to care, but also to strengthen communication, and improve relationships between communities and healthcare institutions, between the federal government and constituents, and between practitioners and patients. Communication needs to be facilitated so that people's needs are heard and respected.

**DR. MADISON:** I have three nieces in the healthcare field. One is a black female physician, Chicago. Another black nurse in the LA area, managing 80 nurses. And the third, black female social worker. They all agreed on their answer to this question, and what they said was, “Let us work towards disease prevention and health promotion instead of a curative model for health care delivery.”

***DR. NÚÑEZ:* One of my favorite acronyms is PDQ, which stands for partner-defined quality. If we can start with the PDQ straight away, then hopefully we can take advantage of this opportunity for good.**

**There is an Asian proverb that says, “The best part of my house burning down is I have a good view of the moon.” In crisis, there is opportunity. We are awash with opportunity. The question, as has been mentioned so eloquently by many of you, is, do we then leverage this opportunity for the better in terms of efforts of equity, relationships, communication, infrastructure, to roll it back resulting in a robust, effective prevention model? We will be able to say, “Yeah, this is not just. We've learned from the Spanish flu. COVID-19 was the tipping point where we changed things up.”**

**I really, really appreciate all of your time. This was a fabulous conversation. It was just really a wonderful opportunity, and thanks so much for all your insights.**

## Moderator


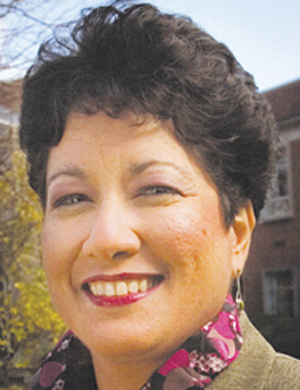


**Ana Núñez, MD**, is a professor of medicine and professor of obstetrics and gynecology, Dean of Diversity, Equity & Inclusion at Drexel University College of Medicine. Her expertise includes sex/gender CBR and health and workforce enhancement for underrepresented populations.

## Participants


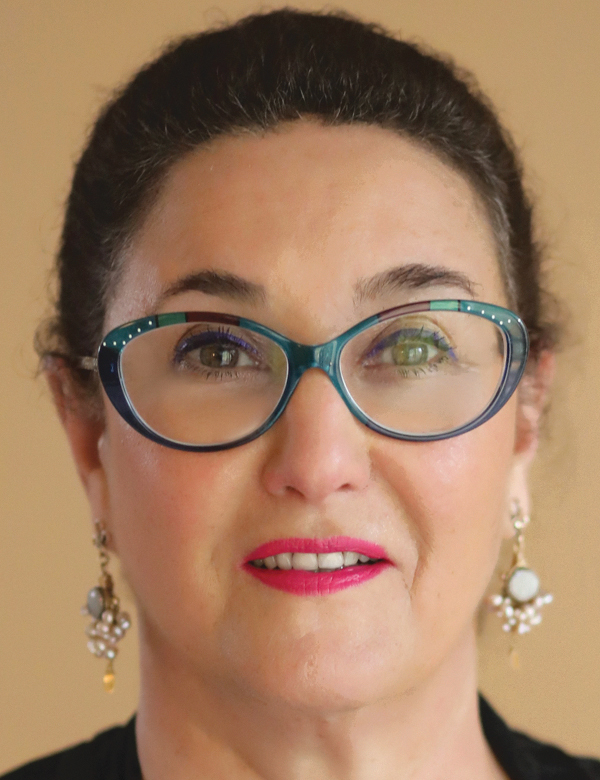


**Ronit Elk, PhD** is Professor, Department of Medicine, Division of Geriatrics, Gerontology and Palliative Care, at the University of Alabama, Birmingham, and Associate Director of the Center of the Southeast Institute for Innovation in Palliative Care. Her research focuses on partnering with underserved communities to develop culturally concordant palliative care interventions, based on the community's cultural values and preferences and at end of life, and in developing effective training methods for clinicians in providing the culturally concordant care. Dr. Elk has published extensively but is most proud of her work as guest Editor of a Special issue in the Journal of Palliative Medicine, focusing on Palliative and End of Life Care for African Americans. The title of her editorial: “The first step is recognizing, acknowledging, and respecting the inequity, disrespect, and disregard our African American patients have experienced.”


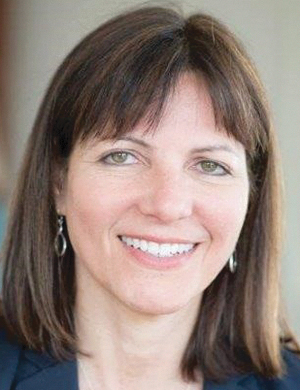


**Holly G. Prigerson** is the Irving Sherwood Wright Endowed Chair of Medicine, Co-Director, Cornell Center for Research on End-of-Life Care, and Professor of Sociology in Medicine at Weill Cornell Medicine. Her research has been continuously funded for over 30 years by the National Institutes of Health to examine issues of health care disparities at the end-of-life and psychosocial influences on and outcomes of those disparities.


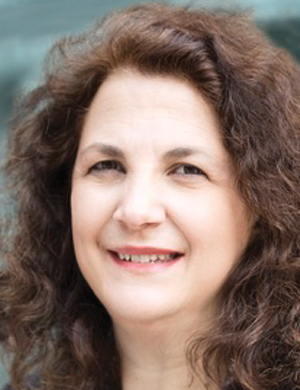


**Renata Schiavo, PhD, MA, CCL,** is a senior lecturer at Columbia University Mailman School of Public Health, Department of Sociomedical Sciences, and the founder and board president of Health Equity Initiative, a nonprofit membership organization. She is a passionate advocate for health equity and a committed voice on the importance of addressing and removing barriers that prevent people from leading healthy and productive lives. She has significant experience with and has written on communicating risk and promoting disease mitigation measures in epidemics and emerging disease outbreak settings.


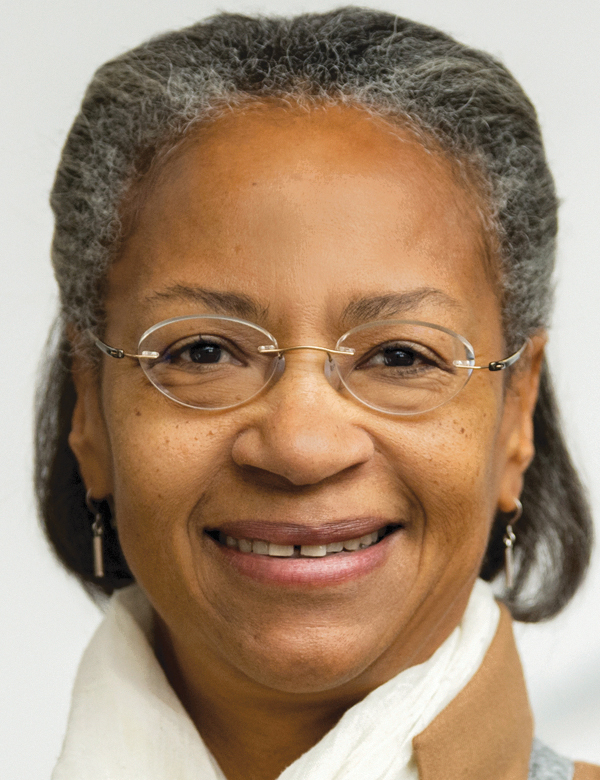


**Dr. Maria Madison** has built her career, since 1983, around evidence-based research methods. This has included conducting and supervising significant public health projects with multicultural communities, often in resource constrained settings. She began her career as a Peace Corps Volunteer in the Democratic Republic of the Congo, (i.e., Zaire), and continued working through the private and public sector. Dr. Madison is currently the Associate Dean for Equity, Inclusion and Diversity at the Heller School for Social Policy and Management at Brandeis University. She teaches on subjects such as Intersectionality and Bioethics.

## Supplementary Material

Supplemental data
