# Supplementary material for: Responding to Healthcare Disparities and Challenges With Access to Care During COVID-19
Source: Health Equity. 2020 Apr 14;4(1):117–28. doi: 10.1089/heq.2020.29000.rtl (PMC7197255; doi:10.1089/heq.2020.29000.rtl)
Supplement: Supplemental data [file Suppl_AppendixS1.pdf]

UNITED STATES of CARE

Playbook
City-Level COVID-19 Rapid Response To Serve At-Risk Populations

Case Study: New York City COVID-19 Rapid Response Coalition
April 2020 Launch

Version 1

The **New York City COVID-19 Rapid Response Coalition** (CRRC) developed a process to serve at-risk populations during the 2020 pandemic.

United States of Care drafted a playbook to highlight replicable approaches, methods, and partnerships that may be valuable in other communities.

Target Audience

If you're in a city with a patchwork system of legacy health care and social service solutions, you are about to face hundreds of thousands of COVID-19 cases.

You will need a creative, impactful way to protect and serve your most vulnerable.

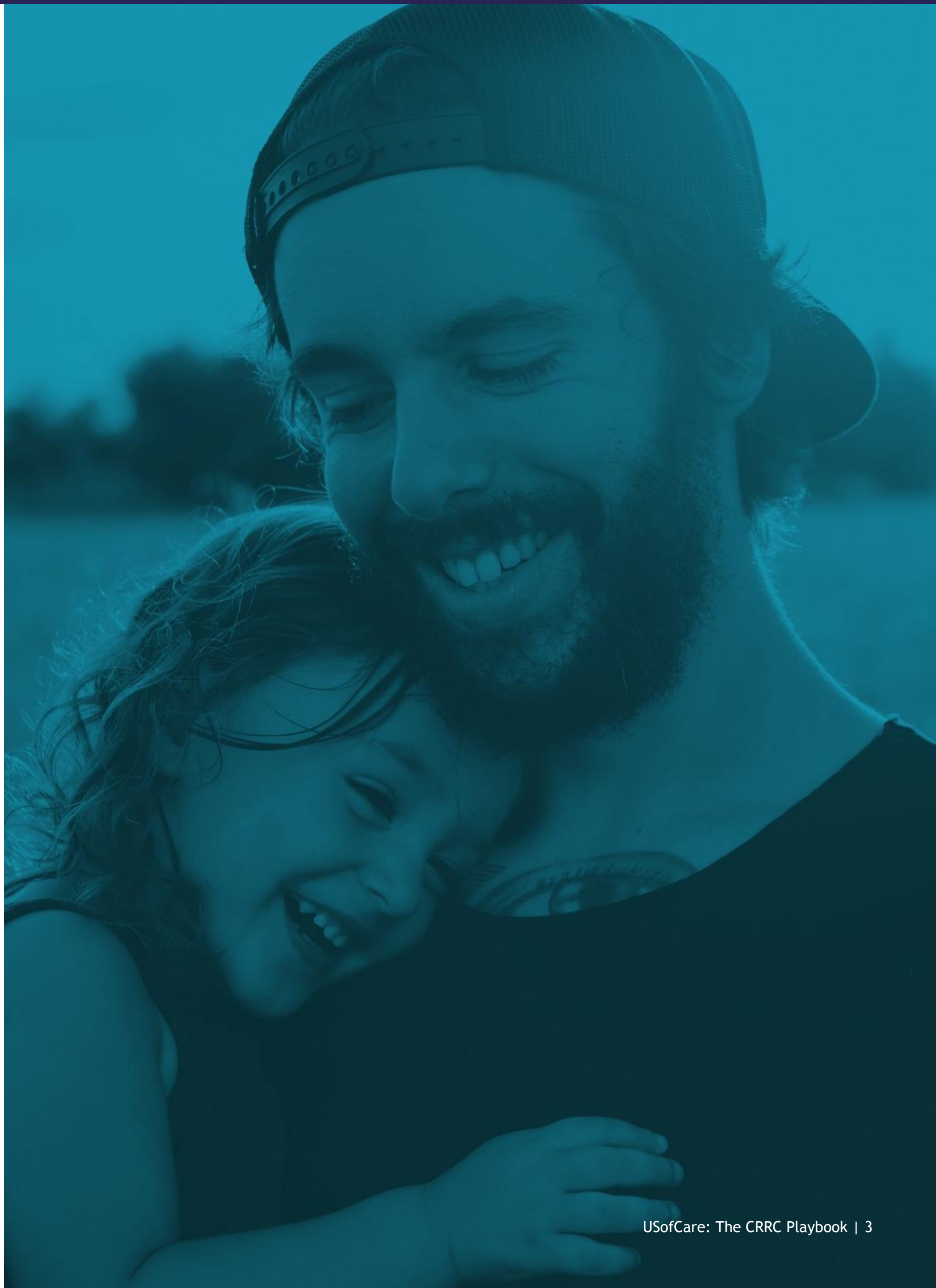

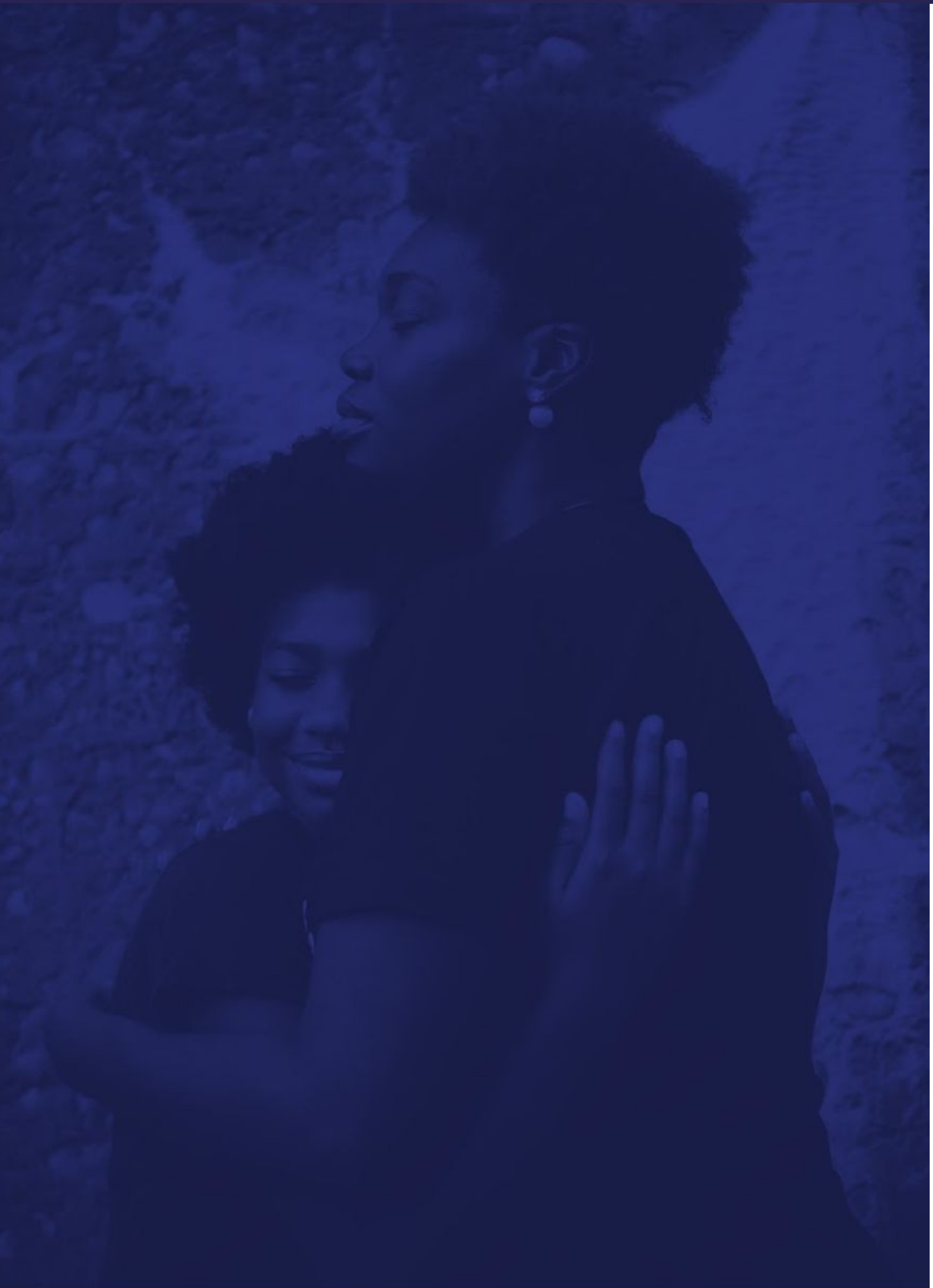

Project Scope

This approach requires the rapid formation of partnerships across multiple, fragmented parts of the health care system.

We've broken down the goal, tactics, and workstreams that the New York City COVID-19 Rapid Response Coalition (CRRC) is using to:

- ★ Connect with & support high-risk community members.
- ★ Facilitate communication across networks, & track patient engagement.
- ★ Identify gaps & match to available services and excess capacity.
- ★ Supplement critical resources in hotspots.

Case Study: New York City COVID-19 Rapid Response Coalition (CRRC)

- Founded in March 2020, organizations and concerned citizens are collaborating to save lives. In a matter of weeks, workstreams and methodologies have been woven together to form the NYC CRRC.

- The following actions are being employed concurrently in this particular case, but it’s not necessary. **Employing any of these tactics should improve outcomes.**

UNITED STATES of CARE

NYC COVID-19 Rapid Response Coalition

Guiding Principles

Humanitarian efforts first.
Pro bono work whenever possible.
Self interest second to communal need.
Profiteering and competition have no place here.
We’re here to help.

★

Goals: What is CRRC solving for?

Saving lives.

1. Caring for and preventing those who are most at risk from contracting COVID-19 and rapidly progressing to the ER/ICU.

2. Helping them avoid acute episodes related to underlying chronic conditions (i.e., stroke, heart attack, respiratory kidney failure).

3. Supplementing care by creating a centralized platform that marries technology and clinical expertise with health care and social service support.

4. Taking pressure off the hospitals and reducing the strain on the city's health care system.

The CRRC Product

- A **dynamic texting platform** that:
  - Allows simple script writing to connect patients with support.
  - Draws from analysis tools and pre-existing data sources.
  - Contacts high-need individuals identified through patient risk assessments, and connects them with telehealth and teleservices.

- A **network of relationships** that weave together:
  - Care provision and support from a coalition of 60+ social and clinical services.
  - Data and analytics from a variety of sources, including health care payers and navigators.

Resulting in **rapidly-scaled communications** that seek and engage high-risk patients, establish needs, and route them to the best service providers with capacity.

CRRC Process and Tech Overview

Convene & Coordinate:
- Facilitate communication across networks & track patient engagement
- Identify gaps and match to available services and excess capacity
- Supplement resources for critical areas
- Develop & disseminate tools & guides to future hotspots

Network Partners
- Validate patient needs
- Screen for clinical, behavioral health and social services
- Coordinate/deliver services

Social Services: *BH, Community Partners*

Clinical Care: *Hospital Systems, PPSs*

Coalition Services (60+ organizations)

TeleServices & TeleHealth

Activate Services

Coordinating Center

Data

Roster

Automated Patient Engagement Platform

Outbound SMS & Calls
Inbound Queries

High Risk Patient Populations

Analytics

Roster

Data Provision:
(Example sources)
- MetroPlus
- HealthFirst
- Affinity
- NYU HEAL NYS Medicaid
- HealthIX
- Other sources

Analysis:
- Apply patient risk assessment and segmentation methodology
- Identify "high-need" patients and likely support requirements
- Create patient rosters with contact information

Engagement Center:
- Proactive & reactive patient engagement: SMS messaging, assisted calling, inbound call management
- Consider build out of a light technology platform to facilitate tracking of capacity and coordination of services (i.e., a virtual marketplace)
- Activate services:
  - Health Screening
  - Telehealth
  - Food & medicine delivery
  - Behavioral Health
  - Other Social Services

The Partnerships

To execute on the process and create a useful product for the people who need it most, relationships must be built across industries and functional capacities.

Success will involve community leaders from a range of sectors, including:

- Lawyers and policy professionals
- Social services professionals
- Clinical professionals familiar with at-risk populations
- Data and analytics professionals
- Project management experts

Case Study: CRRC Partners

Providers

- Maimonides Medical Center
- NYU Langone Health
- Montefiore
- NYC Health + Hospitals
- GNYHA (Greater New York Hospital Association)
- Mount Sinai
- Northwell Health

Payers

- healthfirst – Health Insurance for New Yorkers
- Cigna
- VNSNY (Visiting Nurse Service of New York) CHOICE Health Plans
- EmblemHealth
- aetna

Social Services

- AIR nyc
- UNITE US
- FOOD BANK FOR NEW YORK CITY
- Public Health Solutions
- HSC Human Services Council
- NOWPOW
- Community Healthcare Network

Alternative Care Providers

- CITYBLOCK
- Landmark
- Ready Responders
- CITYMD URGENT CARE
- MJHS HEALTH SYSTEM
- GoHealth URGENT CARE
- RubiconMD
- healthcare Vesta

DME

- integra partners
- adapthealth

Delivery

- Uber
- CAPSULE
- lyft
- CVS Health
- Hello FRESH

Technology

- HealthReveal
- amazon
- TELADOC
- LetsGetChecked
- CRISIS TEXT LINE

Legal/Capital/Project Management

- DEERFIELD
- BAIN & COMPANY
- GENERAL ATLANTIC
- WCAS
- Oxeon
- manatt

The Tactics

CRRC is assessing needs, and matching and meeting them with available capacity.

IDENTIFICATION OF MOST VULNERABLE	Using risk stratification, identify those who are at greatest risk of mortality from COVID-19: those who have pre-existing chronic conditions and whose clinical state will decline rapidly due to constraints on the health care system.
NEED ASSESSMENT AND PATIENT ENGAGEMENT	Engage the patients using scalable technology Assess their needs and educate them on the situation and protective and preventive measures.
CONNECT PATIENTS WITH SERVICES	Based on their digital engagement, connect patients with the various clinical and social services they require. Refer into existing providers and solutions.
MONITOR EFFECTIVENESS AND PRODUCTIVITY	Leverage closed-loop communications to verify that the clinical and social services support is delivered and fulfilled, and that the patients feel supported and safe in their homes and do not require additional hospital-based interaction.

The Tactics

IDENTIFICATION OF MOST VULNERABLE

Using risk stratification, identify those who are at greatest risk of mortality from COVID-19: those who have pre-existing chronic conditions and whose clinical state will decline rapidly due to constraints on the health care system.

- ★ Using claims data, **create patient rosters** with contact information.
- ★ Obtain risk segmented lists and data from payers and providers.
  Note: *The NYC CRRC has received this patient information from MetroPlus, NYC Health and Hospitals, Healthfirst, Mt. Sinai, and Montefiore.*
- ★ Develop and **apply patient risk assessment** and segmentation methodology.
  Note: *The NYC CRRC has identified data and risk analytics vendors to assist with this effort through pro bono contributions, for eventual reimbursement.*
- ★ **Identify "high-need" patients** and their related support needs and requirements.

UNITED STATES of CARE

The Tactics

NEED ASSESSMENT AND PATIENT ENGAGEMENT

Engage the patients using scalable technology. Assess their needs and educate them on the situation and protective and preventive measures.

★ **Screen** for clinical, behavioral health, and social service needs.

★ **Validate** patient needs, and coordinate delivery of services to individuals.
- Build a digital engagement center.
- Consider expanding the platform to facilitate tracking of capacity and coordination of services.

★ **Identify gaps** in services and match individuals to available services, particularly those that have excess capacity, **& scale** using SMS decision trees.
- Decision trees and flow to providers can be customized to either:

  1. Direct the network of providers to their existing network of patients or
  2. Pool providers to address patient surges and critical spikes in need across the system.

★ **Develop & disseminate** tools and guides to patients and service providers in areas that may have high prevalence of infection.

UNITED STATES of CARE

The Tactics

CONNECT PATIENTS WITH SERVICES

Based on their digital engagement, connect patients with the various clinical and social services they require. Refer into existing providers and solutions.

- **Consider daily stand up meetings** to allot time to document areas of need, develop solutions, and foster collaboration across all parties.
- Identify different coalition entities and **crowdsource** to foster group thinking efforts.
- **Develop working teams** and outsource perspectives and services from various parts of the health care industry for comprehensive thought leadership, including:
  - Nonprofit health systems
  - Safety net payers
  - Delivery services
  - Alternative care methods
  - DME/supply experts
  - Medication dissemination
  - Social service providers, including food banks

UNITED STATES of CARE

The Tactics

MONITOR EFFECTIVENESS AND PRODUCTIVITY

Leverage closed-loop communications to verify that the clinical and social services support is delivered and fulfilled, and that the patients feel supported and safe in their homes and do not require additional hospital-based interaction.

★ **Develop a charter** and articulate a clear conflict of interest policy.

★ **Build an operating committee:**

- Provide insight into procedural functioning to assure effectiveness based on past experiences.
- Members of the operating committee should consist of health care leaders representing diverse backgrounds and expertise. Each should have clear participatory roles in:
    - Operations
    - Clinical services
    - Social services
    - Project management

Note: *It may also be beneficial to partner with a local or national consulting firm (i.e. Bain & Company, McKinsey & Company, etc.) that can commit pro bono hours.*

★ **Fundraise & establish an oversight committee:**

- Build a relationship with a reliable funder to support the effort, assuring consistent coverage for those benefiting from the product.
- Members of the oversight committee might include: local health and social services leaders, local business leaders (i.e. PE Partners, CEOs or Managing Partners of Financial Services or Law Firms), and former state and federal government officials.

UNITED STATES of CARE

The Workstreams

After selecting one or more approaches to support your efforts, consider segmenting efforts into different workstreams. CRRC has broken them down into the following:

Partnership Management	Solution Development & Delivery	Assessment of Productivity, Effectiveness, & Need
★ Coalition Management<br>★ Government Affairs<br>★ Fundraising	★ Pilot<br>★ In-person Care Provision<br>★ Virtual Care Provision<br>★ Social Service Support (SSS) Including Food / Supplies Provisioning	★ AWS Closed-Loop Communication Platform<br>★ Population Identification

Partnership Management

★ **Coalition Management**

- Build and manage the coalition of partners required to support and activate a rapid response coalition, including all aspects of project management and communication.

★ **Government Affairs**

- Build and manage relationships with key government partners, including management of all regulatory issues.

★ **Fundraising**

- Secure necessary fundraising, working with range of private/public payers and philanthropic partners.

UNITED STATES of CARE

Solution Development & Delivery

★ Pilot

- Rapidly launch pilot to facilitate provisioning of care, food, supplies,and social services to a limited cohort of at-risk NYC residents.

★ In-person Care Provision

- Identify need for clinical services.
- Identify where clinical capacity exists.
- Ensure match of supply and demand.
- Establish proper decision trees for types of care provision.

★ Virtual Care Provision

- Identify needs for clinical services.
- Identify where clinical capacity exists.
- Ensure appropriate match of supply and demand.

★ Social Service Support (SSS), Including Supplies and Food Provision

- Identify need for SSS services.
- Identify where SSS capacity exists.
- Ensure appropriate match of supply and demand.
- Establish proper decision trees for types of service provision.

Assessment of Productivity, Effectiveness, & Need

★ **AWS Closed-Loop Communication Platform**

- Build platform to manage all patient-facing communication,including workflows for routing patient requests and needs.

★ **Population Identification**

- Identify the most at-risk populations in NYC to address with this program.
- Procure risk-segmented patient data and contact information from payers and providers.
- Use analytics vendors to conduct patient risk assessments.

Use Case

A potential application

User: A safety net health plan affiliated with a safety net hospital that has diminished care management capabilities due to sickness and external distribution of staff to keep the hospital afloat.

Process: The user provides a risk-stratified list of their members with contact information. The CRRC walks through the following:

★ Patient Identification

- 500,000 total Managed Care Medicaid, MLTC, and dual-eligible members.
- 200,000 kids are removed.
- 300,000 adults are assessed, stratifying by age (40+) and those with complex conditions.
- 85,000 members remain.

★ Patient Connection

- Write engagement scripts to be delivered over SMS.
- Test 1,000 reaches and track response rate.
- Allocate responses across the decision tree.
- Fulfill the required service (telemedicine, food supports, et cetera).
- Use SMS to verify they got what they need.

★ Restart Process

- Expand outreach to more members.
- Add new provider and payer data sources.

For More Information, Please Contact

The COVID-19 Rapid Response Coalition: info@NYCCovid19.org

United States of Care: help@usofcare.org

UNITED STATES of CARE
